# Minor Clinical Impact of COVID-19 Pandemic on Patients With Primary Immunodeficiency in Israel****

**DOI:** 10.3389/fimmu.2020.614086

**Published:** 2021-01-14

**Authors:** Nufar Marcus, Shirly Frizinsky, David Hagin, Adi Ovadia, Suhair Hanna, Michael Farkash, Ramit Maoz-Segal, Nancy Agmon-Levin, Arnon Broides, Amit Nahum, Elli Rosenberg, Amir Asher Kuperman, Yael Dinur-Schejter, Yackov Berkun, Ori Toker, Shmuel Goldberg, Ronit Confino-Cohen, Oded Scheuerman, Basel Badarneh, Na‘ama Epstein-Rigbi, Amos Etzioni, Ilan Dalal, Raz Somech

**Affiliations:** ^1^ Allergy and Immunology Unit, Schneider Children's Medical Center of Israel, Felsenstein Medical Research Center, Kipper Institute of Immunology, Petach Tikva, Israel; ^2^ Sackler Faculty of Medicine, Tel Aviv University, Tel Aviv, Israel; ^3^ The Jeffrey Modell Foundation Israeli Network for Primary Immunodeficiency, New York, NY, United States; ^4^ Pediatric Department A and the Immunology Service, Jeffrey Modell Foundation Center, “Edmond and Lily Safra” Children‘s Hospital, Sheba Medical Center, Affiliated to the Sackler Faculty of Medicine, Tel Aviv University, Tel-Aviv, Israel; ^5^ Clinical Immunology, Angioedema and Allergy Unit, Center for Autoimmune Diseases, Sheba Medical Center, Tel Hashomer, Israel; ^6^ Sackler School of Medicine, Tel Aviv University, Ramat-Aviv, Israel; ^7^ Department of Medicine, Allergy and Clinical Immunology Unit, Tel Aviv Sourasky Medical Center, Tel Aviv, Israel; ^8^ Pediatric Allergy Unit, E. Wolfson Medical Center, Holon, Israel; ^9^ Pediatric Department, E. Wolfson Medical Center, Holon, Israel; ^10^ Ruth Children Hospital, Rappaport Faculty of Medicine, Technion, Haifa, Israel; ^11^ Immunology Clinic, Soroka University Medical Center, Faculty of Health Sciences, Ben-Gurion University of the Negev, Beer Sheva, Israel; ^12^ Azrieli Faculty of Medicine, Bar-Ilan University, Safed, Israel; ^13^ Blood Coagulation Service and Pediatric Hematology Clinic, Galilee Medical Center, Nahariya, Israel; ^14^ Bone Marrow Transplantation Department, Hadassah-Hebrew University Medical Center, Jerusalem, Israel; ^15^ Department of Pediatrics, Mount Scopus Hadassah-Hebrew University Medical Center, Jerusalem, Israel; ^16^ Faculty of Medicine, Hebrew University of Jerusalem, Jerusalem, Israel; ^17^ The Allergy and Immunology Unit, Shaare Zedek Medical Center, Jerusalem, Israel; ^18^ Pediatric Pulmonary Unit, Pediatric Division, Shaare Zedek Medical Center, Jerusalem, Israel; ^19^ Allergy and Clinical Immunology Unit, Meir Medical Center, Kfar-Saba, Israel; ^20^ Pediatrics B, Schneider Children Medical Center Israel, Sackler School of Medicine, Tel Aviv University, Tel Aviv, Israel; ^21^ Pediatric Department, Allergy and Immunology Clinic, Carmel Medical Center, Technion Faculty of Medicine, Haifa, Israel; ^22^ Institute of Allergy, Immunology and Pediatric Pulmonology, Shamir (Former Assaf Harofeh) Medical Center, Zerifin, Israel

**Keywords:** pandemic, severe acute respiratory syndrome-coronavirus-2, SARS-CoV-2, agammaglobulinemia, inborn errors of immunity, primary immunodeficiency, coronavirus disease 2019, COVID-19

## Abstract

In the last few months the world has witnessed a global pandemic due to severe acute respiratory syndrome-coronavirus-2 (SARS-CoV-2) infection causing coronavirus disease 2019 (COVID-19). Obviously, this pandemic affected individuals differently, with a significant impact on populations considered to be at high-risk. One such population, was assumed to be patients with primary genetic defect involving components or pathways of the immune system. While human immunity against COVID-19 is not fully understood, it is, so far, well documented, that both adaptive and innate cells have a critical role in protection against SARS-CoV-2. Here, we aimed to summarize the clinical and laboratory data on primary immunodeficiency (PID) patients in Israel, who were tested positive for SARS-CoV-2, in order to estimate the impact of COVID-19 on such patients. Data was collected from mid-February to end-September. During this time Israel experienced two “waves” of COVID-19 diseases; the first, from mid-February to mid-May and the second from mid-June and still ongoing at the end of data collection. A total of 20 PID patients, aged 4 months to 60 years, were tested positive for SARS-CoV-2, all but one, were detected during the second wave. Fourteen of the patients were on routine monthly IVIG replacement therapy at the time of virus detection. None of the patients displayed severe illness and none required hospitalization; moreover, 7/20 patients were completely asymptomatic. Possible explanations for the minimal clinical impact of COVID-19 pandemic observed in our PID patients include high level of awareness, extra-precautions, and even self-isolation. It is also possible that only specific immune pathways (e.g. type I interferon signaling), may increase the risk for a more severe course of disease and these are not affected in many of the PID patients. In some cases, lack of an immune response actually may be a protective measure against the development of COVID-19 sequelae.

## Introduction

The latest outbreak of severe acute respiratory syndrome coronavirus 2 (SARS-CoV-2) has resulted in a global pandemic, which was named coronavirus disease 2019 (COVID-19), affecting so far more than 33 million people worldwide, with an overall death toll of more than a million (~2.99%) (1). The clinical spectrum of COVID-19 is extremely variable, ranging from an asymptomatic state to a severe respiratory distress syndrome. Many acute extra-pulmonary complications as well as late manifestations including hyperactive immune response are constantly being reported (2). While several comorbidities such as older age, male gender, hypertension, diabetes, and obesity were identified as risk factors predisposing individuals to a severe disease (3), many individuals, who were considered “healthy,” suffered also from an unfavorable outcome. The poor outcome of those who lack overt risk factors could possibly be attributed to several causes including “virus related factors,” such as infection with a larger viral inoculum, or a more virulent SARS-CoV-2 strains, environmental conditions, and “host dependent factors,” such as inevitable acquired somatic and epigenetic changes occurring over time, or congenital monogenic predisposition to SARS-CoV-2 due to a genetic defect affecting one or more components of the immune system (primary immunodeficiencies or inborn errors of immunity) (4). Primary immunodeficiency (PIDs) is a group of genetic disorders characterized by an impaired host defense, resulting in an increased susceptibility to infections, inflammation, autoimmunity, allergy, and cancer. The incidence of these diseases, ranges from 1:500 to 1:1,000,000, depending on the specific primary genetic defect. With the exception of selective immunoglobulin A (IgA) deficiency, which is relatively common in the general population, the overall estimated prevalence of these disorders is approximately 1 in 1,200 live births (5, 6). In these disorders, different elements of the immune system including cellular (T cells) and/or humoral (B cells) compartments, neutrophils or innate cells and complement components, are disrupted. As depicted from the role of innate immune cells and specific immune pathways (e.g. nucleic acid sensor molecules or IFN pathway) in protection against certain viruses, it is predicted that a normal immune response to SARS-CoV-2 also depends on innate immunity. It is not surprising therefore that a monogenic defect that predisposed individuals uniquely to severe COVID-19 disease was linked to the TLR7 gene (7). Recently other variants of genes involved in innate immune pathways were suggested to underlie a severe clinical course of COVID-19 in 23 critically ill patients (8). These include autosomal-recessive (AR) deficiencies (IRF7 and IFNAR1) and autosomal-dominant (AD) deficiencies (TLR3, UNC93B1, TICAM1, TBK1, IRF3, IRF7, IFNAR1, and IFNAR2) in 4 and 19 patients, respectively. Furthermore, findings regarding acquired autoantibodies against type 1 INF, have also been linked to a severe disease course (9). However, to date, despite the immense worldwide scientific effort, the full spectrum of human immunity against COVID-19 is not well understood (10). While humoral and cellular immune responses are usually mounted against the virus, hyper immune responses to SARS-CoV2 sometimes results in inflammatory tissue damage, leading to a severe disease and death. Thus, paradoxically, the mortality rate associated with COVID-19 is mostly the result of a dysregulated inflammatory response rather than direct viral injury, or viral replication itself (11). Taken together, an orchestrated innate and adaptive immune response should be able to effectively control SARS-CoV-2 infection without the negative side effects of a hyper immune response (12). Accordingly, it will be reasonable to assume that primary immunodeficiency disorders should be considered another risk factor for developing severe COVID-19.

Israel with a population of about 9 million citizens, had so far lower COVID-19 related mortality compared to global numbers. As of end of September 2020, 233,000 cases were diagnosed with 1,500 deaths (0.65%) reported. In addition, Israel has a high prevalence of primary immunodeficiencies disorders, due to a high rate of consanguine families. For example, for severe combined immunodeficiency (SCID), the most severe type of immunodeficiency that involves both cellular and humoral arms of the immune system, the incidence in Israel is 1:25,000 compared to 1:60,000 in the USA (13, 14). Moreover, in certain consanguineous populations in Israel, the prevalence of any type of primary immunodeficiency is significantly higher (15). Thus, estimating the impact of COVID-19 infection on PID patients in such population is of interest. In this report, we aimed to collect demographic and clinical data on PID patients in Israel who were tested positive for COVID 19 in order to estimate the clinical impact of this disease on patients with primary immune defects.

## Methods

### Patients and PID Centers Enrolment

In order to assess the clinical course of COVID-19 infection in patients with PID, a retrospective survey was conducted. Patients from Israeli PID Centers were enrolled. The diagnosis and management of PID patients in Israel is well organized under the Israel Association of Allergy and Clinical Immunology (IAACI), the Israeli PID society and the Jeffrey Modell Foundation (JMF) network. There are 10 medical centers who follow and treat ~1,700 PID patients in Israel. Centers are led by expert immunologists coordinating and exchanging data on a routine basis. All contributed data to complete this survey. These centers cover the entire population of Israel and nearly all the PID patients in Israel, are followed by specialists form these centers.

Each center provided data on the total PID patients under its care, and the total number of patients (as far as they know) who were tested positive for SARS-CoV-2. Data was collected from mid-February to mid-September. During this time Israel experienced two “waves” of COVID-19 diseases (the first, from mid-February to mid-May and the second began in mid-June and is still ongoing at the end date of data collection). Demographical and clinical data including age, PID type, and underlying genetic cause if known, COVID-19 presentation and diagnosis, treatment, and outcomes were collected from all PID patients who were tested positive for SARS-CoV-2 during the study period. PID was defined according to current diagnostic guidelines and classification of the International Union of Immunological Societies (IUIS) Committee for Inborn Errors of Immunity (16). The study was approved by the Sheba Medical Center and the Schneider Children’s Medical Center and Rabin campus IRB committees.

### SARS-CoV-2 Detection

In 19 of the 20 reported cases, SARS-CoV-2 infection was confirmed by Real-Time Reverse Transcriptase–Polymerase Chain Reaction (PCR) assay of nasal and pharyngeal swabs. Only few patients were re-tested every few weeks. A cut off of below cycles 37 is considered positive according to the Israeli Ministry of Health guidelines. In one patient (Pt 10), the diagnosis was presumptive, based on a known clinical exposure to a positive household contact and suggestive clinical symptoms of pneumonia.

## Results

Ten immunology centers that follow ~1,700 PID patients, provided data for this study, of them, PID patients infected by SARS-CoV-2 were found in seven centers. Three centers reported that they did not encounter any PID patient infected with SARS-CoV-2. All together, we summarized results of 20 PID patients. Their age ranged of 4 months to 60 years. The cohort includes 12 (60%) male patients and 8 (40%) female patients. Immunodeficiency disorders were diagnosed based on IUIS PID classification (16). An underlying genetic defect was known in 15/20 (75%) patients. Sixteen patients (80%) had some degree of humoral immunodeficiency (agammaglobulinemia, hypogammaglobulinemia, or dis-gammaglobulinemia), or a combined immunodeficiency, and were therefore already on monthly Intravenous immunoglobulin (IVIG) replacement therapy prior to the time of SARS-CoV-2 detection. In addition, nine patients were on antibiotic prophylaxis. Chronic lung disease was documented in 11 patients. **Table 1** summarized demographic data and pre COVID-19 clinical status.

**Table 1:** Demographic and clinical data of 20 patients with Primary immunodeficiency before positive SARS-CoV-2 test.

Pt #	Age(years)	Gender	Underling diagnosis	Background lung disease	Other medical problems	IVIG-RT	Other treatment
1	24	F	SCID -RAG2	Bronchiectasis	Irritable bowel syndrome, Vitiligo, Lymphopenia Low TRECs level	Yes	SMX/TMP 20 years post hematopoietic stem cell transplantation
2	30	M	X-HIGM- CD40 Ligand Deficiency	Chronic cough	Hypogammaglobulinemia CD4 lymphopenia	Yes	None
3	17	M	XL -HIGM - CD40 Ligand Deficiency	Chronic cough	Hypogammaglobulinemia	Yes	None
4	19	M	HIGM	None	None	Yes	None
5	17	M	HIGM	None	None	Yes	None
6	6	M	XLA	None	Agammaglobulinemia	Yes	None
7	5	M	XLA	None	Agammaglobulinemia	Yes	None
8	37	M	CVID	Bronchiectasis	Hypogammaglobulinemia	Yes	Amoxicillin Bronchodilators Inhaled corticosteroids Iron supplements
9	22	F	CVID	None	Intestinal lymphonodular hyperplasia	Yes	None
10	60	M	CVID	None	Anemia, Diarrhea	Yes	None
11	30	M	CVID	Bronchiectasis	Celiac like enteropathy, Thrombocytopenia	Yes	Antibiotics Bronchodilators Inhaled corticosteroids
12	59	F	CID- LRBA	Interstitial lung disease	Portal Hypertension Granulomatous disease Anemia	Yes	Azithromycin, Levofloxacin Vitamin D Calcium supplements Bronchodilators Inhaled corticosteroids
13	20	F	CID-RelB Mutation	Bronchiectasis	Dysgammaglobulinemia Celiac enteropathy Autoimmune hemolytic anemia	Yes	None
14	15	F	CID-RelB Mutation	Bronchiectasis	Dysgammaglobulinemia	Yes	None
15	0.3	F	CID-RelB Mutation	None	None—diagnosed genetically due to family history	No	None
16	59	F	CID- CTLA4 Haploinsufficiency	Granulomatous Lung Disease Bronchiectasis	Anemia (nonimmune) Bone marrow lymphocytic infiltrate Splenomegaly Hepatic Lymphocytic infiltrate Portal hypertension Ascites	Yes	Prednisone Azithromycin Vitamin D Calcium supplements
17	33	F	ALPS-like	None	Autoimmune cytopenia Lymphoproliferation Osteoporosis Diabetes Obesity	No	Rapamycin Coumadin Glucophage Vitamin D Calcium supplements
18	16	M	CGD- p47 Mutation	None	Perianal abscess Immune thrombocytopenic purpura Short stature	No	SMX/TMP Itraconazole
19	15	M	CGD- p91 Mutation	None	None	No	SMX/TMP Itraconazole
20	1.5	M	22q11.2 deletion syndrome	Bronchiectasis	Hypogammaglobulinemia Low TRECs Level	Yes	Amoxicillin

IUIS, International Union of Immunological Societies; IVIG-RT, IVIG Replacement therapy; SCID, Severe combined immunodeficiency; RAG-2, recombination-activating gene 2; X-HIGM, X-linked Hyper IgM syndrome; XLA-BTK, X-linked Agammaglobulinemia; BTK, Bruton tyrosine kinase; CVID, common variable immunodeficiency; CID, Combined immunodeficiency; LRBA, lipopolysaccharide (LPS)-responsive and beige-like anchor protein; CTLA4, cytotoxic T-lymphocyte-associated protein 4; ALPS, autoimmune lymphoproliferative syndrome; CGD, Chronic Granulomatous Disease; TRECs, T-cell receptor excision circles; SMX/TMP, Trimethoprim/sulfamethoxazole; NA, not available.

Six patients (30%) were completely asymptomatic and were diagnosed following exposure to a confirmed close symptomatic contact who was tested positive for SARS-CoV-2. Thirteen patients had symptoms commonly associated with COVID-19 infection including: fever, cough, shortness of breath, fatigue, weakness, myalgia, ageusia and anosmia, and abdominal pain. Only one patient was diagnosed with pneumonia. All patients were diagnosed with a mild disease, according to the acceptable disease severity criteria (17). None of our patients suffered from hypoxemia, and none of them required hospital admission. Symptom duration ranged from 1 to 14 days. In most patients, COVID-19 infection resolved without any specific treatment. One patient (Pt 3) with underlying Hyper IgM syndrome (HIGM), received an additional IVIG dose. A second HIGM patient (Pt 2) complained of shortness of breath and was treated empirically with a 3-day course of oral dexamethasone (6 mg/day) which was discontinued after resolution of symptoms. Two additional patients received Azithromycin. Four patients had serial COVID-19 RT-PCR testing, showing resolution in 1–6 weeks. However, this data was not available for the majority of the patients. Other implications of COVID-19 for these patients included prolonged isolation, and for two patients the monthly IVIG replacement therapy was delayed in 1–2 weeks due to hospital infection control reasons. The clinical course of COVID-19 in our patients is summarized in **Table 2**.

**Table 2:** Clinical data and disease course of SARS-CoV-2 positive test and COVID-19 infection in 20 patients with Primary immunodeficiency.

Pt #	Age	Gender	Indication for COVID-19 Testing		COVID-19 Related Symptoms	Symptoms Duration (Days)	Need for Hospitalization	COVID-19 Related Treatment	Change in Basic Treatment	Disease Severity	Other Implications	Positive PCR Duration (weeks)
1	24	F	Exposure to a positive COVID-19 contact		Myalgia	5	No	Azithromycin	None	Mild	None	NA
2	30	M	Fever		Cough, fever, shortness of breath	5	No	Dexamethasone	None	Mild	Delay in IVIG-RT Prolonged isolation	4
3	17	M	Fever, Headache		Fatigue	3	No	IVIG-RT	None	Mild	None	NA
4	19	M	Exposure to a positive COVID-19 contact		Fever, anosmia, myalgia	2	No	None	None	Mild	None	NA
5	17	M	Exposure to a positive COVID-19 contact		Headache, weakness	4	No	None	None	Mild	None	NA
6	6	M	Exposure to a positive COVID-19 contact		Fever	1	No	None	None	Mild	None	NA
7	5	M	Exposure to a positive COVID-19 contact		Asymptomatic	0	No	None	None	None	None	NA
8	37	M	Exposure to a positive COVID-19 contact		Asymptomatic	0	No	None	NA	None	None	NA
9	22	F	Exposure to a positive COVID-19 contact		Fatigue, abdominal pain	60	No	Antibiotic Prophylaxis	None	Mild	Prolonged isolation	6
10	60	M	Fever, cough, Shortness of breath		Pneumonia	7	No	Antibiotic	None	Mild	None	NA
11	30	M	Fever		Cough	5	No	None	None	Mild	Delay in IVIG-RT	3
12	59	F	Exposure to a positive COVID-19 contact		Asymptomatic	0	No	None	None	None	None	NA
13	20	F	Fever		Fever	1	No	None	None	None	None	NA
14	15	F	Exposure to a positive COVID-19 contact	Asymptomatic		0	No	None	None	None	None	NA
15	0.3	F	Exposure to a positive COVID-19 contact	Fever		2	No	None	None	Mild	None	NA
16	59	F	Cough	Insomnia		4	No	None	None	Mild	None	NA
17	33	F	Weakness	Ageusia and anosmia		2	No	None	None	Mild	None	2
18	16	M	Cough, fever	Pneumonia		7	No	Antibiotic	None	Mild	None	1
19	15	M	Exposure to a positive COVID-19 contact	Asymptomatic		0	No	None	None	None	None	NA
20	1.5	M	Exposure to a positive COVID-19 contact	Asymptomatic		0	No	None	None	None	None	NA

IVIG-RT, Intravenous immunoglobulin replacement therapy; NA, Not available.

## Discussion

The impact of COVID-19 pandemic on individuals and populations varies greatly, and higher impact is expected in high-risk patients. In Israel, with approximately 9,000,000 citizens, ~2.5% of the population were tested positive, so far, for SARS-CoV-2 infection (RT-PCR confirmed) (1). Interestingly, out of the 1,679 PID patients that are followed in 10 Israeli PID Centers, only 20 patients (1.2%) were tested positive to SARS-CoV-2. Obviously, it is possible that this number could be higher since not all PID patients were screened, only those who were symptomatic or who had a clear indication for testing. Of note, none of these positive patients showed any significant symptoms or signs, and none required hospitalization. This is in contrast to the general population and raises a question of whether PID is a predisposing or, paradoxically, a protective factor for severe SARS-CoV-2 infection (18, 19). Very few reports on the impact of COVID-19 on PID patients were published so far. One meta-analysis that included eight studies showed that nonspecific immunosuppression and immunodeficiency are associated with the increased risk of severe COVID- 19 disease, although the statistical differences were not significant. For immunodeficiency, only a 1.55-fold increased risk of severe COVID-19 disease, was found. Interestingly, several studies showed that even patients with cancer were not at greater risk to have significant COVID-19 disease (19). Considering more specific PIDs, Quinti et al., reported that agammaglobulinemic patients showed milder course of disease compared to patients with common variable immunodeficiency (CVID), suggesting a role for B cells in disease pathogenesis (20). This was similar to a report of two XLA patients who recovered from SARS-CoV-2 infection despite developing pneumonia (21). Similarly, a CVID patient treated with intravenous immunoglobulin (IVIG) replacement fully recovered from the severe disease despite being considered at increased risk (22). An international survey which was undertaken between March 16 and June 20, 2020, gathered information on 94 patients with PID (23). The median age group was 25–34 years. Patient’s baseline diagnosis was mainly primary antibody deficiency (56%). In contrast to our cohort, the North American and European patients had a more severe disease course. About 2/3 of them were hospitalized, 1/3 of patients (32.9%) required respiratory support (non-invasive or invasive), three patients required extracorporeal oxygenation, and nine patients including two children, succumbed to SARS-CoV-2 infection (10% mortality rate). Nevertheless, the authors concluded that risk factors predisposing PID patients to severe disease and mortality were similar to the general population, although younger male patients were more likely to develop severe COVID-19 and require ICU admission (23). Our national study, while limited in numbers due to the relatively small population of Israel, was done through a non-biased national survey, including all PID patients in Israel. It revealed a low incidence of SARS-CoV-2 infection among PID patients, all with a favorable course and outcome. None of our patients displayed significant clinical symptoms such as hypoxemia, or required hospital admission. In terms of their PID, most (80%) had humoral immunodeficiency or a combined immunodeficiency. This finding was in accordance to the North American and European cohort (23), and therefore were already on IVIG replacement therapy.

Several explanations for lower incidence of COVID-19 in PID patients can be provided. A third of our patients were asymptomatic and were only tested due to exposure to a close positive contact. We can assume that other asymptomatic cases could have been similarly positive through a general mass-screening. On the other hand, we can also assume that the additional precaution measures taken by PID patients led them to perform more tests than the general population and yet they remained negative. PID patients strictly follow precaution recommendations and use additional measures in an attempt to prevent infections. In addition, PID patients are well experienced with hand washing and overall hygiene, mask wearing and physical/social distancing measures. Some even chose self-isolation, reducing to minimum their chances to become infected. An additional explanation, more specific to Israel, could be general government-guided avoidance measures. Until recently, the relative number of positive cases in Israel was lower compared to the rest of the world, which could also explain the low number of positive cases among patients with PID. Most (19/20) of the patients in our cohort were diagnosed during the second wave of COVID-19 in Israel. In that regard, it should be mentioned that severe isolation measures were taken in Israel by all the population during the first wave, with specific additional precaution recommendation to at risk populations (including PID patients). A general lockdown was enforced, schools were closed and work attendance was limited. These measures changed during the second wave when most of our patients went back to work, daycare, and schools. As a result, the general number of COVID-19 cases in Israel dramatically increased. This would explain the higher incidence of COVID-19 during the second wave, but should not affect disease outcome. Whether the fact that many PID patients were on IVIG replacement therapy at the time of infection, may have had a protective effect is yet to be proven, and there is no evidence-based data to support this. Yet it is of note, that several SARS-CoV-2 infected XLA patients hospitalized for COVID-19 showed rapid improvement after infusion of COVID-19 convalescent plasma (24, 25). Interestingly, while analyzing our data, we found a family of four siblings with XLA patients that had a close contact with one of their healthy siblings who came over and stayed with them over the weekend when he developed symptoms of COVID-19. They were all brought into the hospital despite being in isolation to receive their monthly IVIG infusion directly following this exposure. None of the four patients developed any symptoms and all tested negative for COVID-19. The fact that PID patients did not get infected with COVID-19 despite the fact that other household members were actively ill has been reported by several centers in Israel, but this interesting point is beyond the scope of this publication.

PID is a heterogeneous group of diseases that affect many components of the immune system, not all of them are relevant for the protection against COVID-19. For example, patients with complement deficiency or auto-inflammatory syndromes may have no influence on the ability to mount protection against COVID-19. Patients with chronic arthritis and auto inflammatory syndromes either treated with Disease-modifying antirheumatic drugs (DMARDs) or not, were found not to be at an increased risk of respiratory or life-threatening complications from SARS-CoV-2 (26). As such, FMF which is an autoinflammatory disease with high prevalence in Israel but with no known predisposition to COVID-19 illness was excluded from our survey. Some even suggested a protective effect to colchicine treatment that is the standard of care for many Familial Mediterranean Fever patients (27). Thus, not all PID patients should be included when trying to estimate the true impact of COVID-19 on PID patients. Our survey focused on PIDs which mainly predispose to infections including: cellular, humoral, combined, and phagocyte immunodeficiency. In addition, the younger age of many of the PID patients could be another protective factor as it is well documented that clinical disease and unfavorable outcome are rare in younger age (28).

While it is appropriate to assume that normal cellular and humoral immunity are enough for virus suppression, it is well accepted now that the pathophysiology underlying the clinical manifestations of COVID-19 is less influenced by the immune status of an individual but is chiefly related to the direct cytopathic effects of SARS-CoV-2 on respiratory epithelia, endothelia, and other organ-specific cell types, mounting a pro-inflammatory cytokines secretion and dysregulated adaptive immunity leading to tissue damage (29). It is now clear that non-genetic causes resulting in overexpression of Angiotensin-converting enzyme 2 (ACE2) has had a critical role in the severity of the disease. ACE2 is the common factor that binds to the superficial S glycoprotein on the viral envelope (30). It is possible that this binding regulated by TLR7, presented in endosomes leading to an increase secretion of inflammatory cytokines. Caused by defined monogenic defects, PIDs help in shedding light on mechanisms and immune pathways central for the control of specific pathogens. Accordingly, mutations in *TLR7* were reported as a predisposing factor to acquire severe COVID-19 disease (7). More recently, other defects in nucleic acid sensory molecule and type I interferon (IFN) signaling and amplification were identified in 3.5% percent of severely affected patients with no prior severe infectious history. In these patients, enrichment of rare variants predicted to be loss-of-function at the 13 human loci known to govern TLR3- and IRF7-dependent type I interferon (IFN) immunity, was reported. This suggests that inborn errors of TLR3- and IRF7-dependent type I IFN immunity can underlie life-threatening COVID-19 (8). Moreover, neutralizing auto-antibodies against different Type I IFNs in more than 10% of patients with life-threatening COVID-19, were detected, reinforcing the role of IFNs in controlling SRAS-CoV-2 infection (9). On the other hand, it is possible that some PID defects offer a protection against elements in the disease pathogenesis, including the development of the hyper immune reaction. For example, BTK deficiency is considered an advantage and B-cell deficiency was suggested to prevent inflammation, a major devastating sequela of the disease.

This observational study presents several limitations. As mentioned earlier this was a relatively small cohort of only 20 patients, which makes it difficult to draw a statistically significant based conclusion. It is possible that other PID patients could have been positive for SARS-CoV-2, yet were asymptomatic and therefore were not tested. We believe that most if not all PID patients in Israel are followed by one of the 10 centers who contributed data for this study. Obviously, PID patient with severe COVID-19 disease would be brought to our attention. Another important limitation was the fact that repeated PCR testing, for the detection of SARS-CoV-2, was not done for most of patients, therefore we were unable to explore a long-term carrier status, as previously suggested. The strength of this study is the fact that it is a national study, including most of known PID patients in a country that has a relatively high prevalence of PIDs. The fact that patients with PID displayed a relatively a mild course of COVID-19 disease is clearly important for PID patients, who previously adhered to strict isolation, and in light of this data may consider resuming work or school attendance with precautions. As the pandemic course continues to be active worldwide this is of great importance in allowing our patients to function through these stressful times. Our results should be taken very cautiously, as they may be relevant to a specific patient population, environment, health system setup, and time. Israel is now experiencing a surge of SARS-CoV-2 positive cases and the number of affected patients constantly growing. Thus, it is possible that this surge will be followed by a higher number of PID patients with more severe COVID-19. Finally, high level of awareness, strict adherence to hygiene and social distancing guidelines, and continuation of routine treatment and follow-up could all help reducing the incidence of COVID-19 in our patient population and potentially affect disease severity. It is reasonable to assume that other genetic defects will be discovered soon as new inborn errors of immunity altering human protection against SARS-CoV-2.

## Data Availability Statement

The original contributions presented in the study are included in the article/supplementary material. Further inquiries can be directed to the corresponding author.

## Ethics Statement

The studies involving human participants were reviewed and approved by the Sheba Medical Center and the Schneider Children’s Medical Center and Rabin campus IRB committees. Written informed consent from the participants’ legal guardian/next of kin was not required to participate in this study in accordance with the national legislation and the institutional requirements.

## Author Contributions

NM, SF, and RS followed the patients, conceptualized the study, supervised results, and wrote the manuscript; DH followed the patients, provided valuable clinical data, and assisted in writing the manuscript; AO, SH, MF, RM-S, NA-L, AB, AN, ER, AK, YD-S, YB, OT, SG, RC-C, OS, BB, NE-R, AE, and ID followed the patients and provided valuable clinical data. All authors contributed to the article and approved the submitted version.

## Conflict of Interest

The authors declare that the research was conducted in the absence of any commercial or financial relationships that could be construed as a potential conflict of interest.

## Abbreviations

CVID, common variable immunodeficiency; COVID-19, coronavirus disease 2019; PID, primary immunodeficiency disorders; SARS-CoV-2, severe acute respiratory syndrome-coronavirus-2; TLR, toll like receptor.
